# Long-term outcomes after previable pPROM: implications for counselling at the limit of viability

**DOI:** 10.1007/s00431-026-07266-x

**Published:** 2026-07-30

**Authors:** Agnes Grill, Renate Fuiko, Raphaela Pichler, Sophie Jansen, Fanny Mikula, Alex Farr, Angelika Berger, Katharina Goeral

**Affiliations:** 1 Division of Neonatology, Intensive Care and Neuropediatrics, Department of Pediatrics and Adolescent Medicine, Comprehensive Center for Pediatrics, Medical University of Vienna, Vienna, Austria; 2 Division of Obstetrics and Feto-Maternal Medicine, Department of Obstetrics and Gynecology, Comprehensive Center for Pediatrics, Medical University of Vienna, Vienna, Austria

**Keywords:** Preterm prelabour rupture of membranes, Previable pPROM, Limit of viability, Extreme prematurity, Survival, Neurodevelopmental outcome

## Abstract

The purpose of this study is to provide institution-specific, contemporary long-term outcome data to improve counselling at the limit of viability after previable preterm prelabour rupture of membranes (pPROM). This is a retrospective cohort study (2009–2022) of infants with pPROM < 23 + 0 weeks’ gestation who received active neonatal care. Primary outcome was neurodevelopmental outcome at 24–36 months’ corrected age. Among 109 infants, 33 (30.3%) died before discharge. Of 76 survivors, 13 (17.1%) were lost to follow-up and outcome data were available for 63/76 (82.9% follow-up). The mean gestational age was 21.6 weeks at pPROM and 25.7 weeks at delivery. Median Bayley-III scores were 90 (IQR 73–100) for cognition, 81 (IQR 63–97) for language, and 89 (IQR 65–100) for motor function. Cerebral palsy occurred in 11.1%. Survival without moderate or severe neurodevelopmental impairment (NDI) was 72.6%; moderate NDI occurred in 11.0% and severe NDI in 16.4%. Latency duration and gestational age at rupture were not significantly associated with outcome. Severe neonatal morbidity was associated with lower survival without moderate or severe NDI (57.6% vs. 89.7%). In multivariable analysis, gestational age at birth and female sex were associated with favourable outcomes in a perinatal model; after inclusion of major neonatal morbidities, only morbidity burden remained independently associated with outcome (OR 0.39, 95% CI 0.17–0.89).

*Conclusion*: A substantial proportion of survivors after pPROM < 23 weeks achieved favourable neurodevelopment at 2–3 years. In this cohort, latency duration and gestational age at membrane rupture were not independently associated with long-term neurodevelopmental outcome. However, any influence of antenatal factors may be mediated through neonatal morbidity. These findings support individualized counselling that considers both antenatal factors and the subsequent postnatal clinical course when discussing long-term prognosis.

**Table:** 

**What is Known:** • *Previable preterm prelabour rupture of membranes (pPROM) before 23 weeks’ gestation is associated with high perinatal mortality and substantial risk of long-term neurodevelopmental impairment, but available follow-up data remain limited.* • *Previous studies have suggested that gestational age at membrane rupture and latency duration may influence survival, yet their association with long-term neurodevelopment among survivors remains unclear.*	
**What is New:** • *In this single-centre cohort with standardized Bayley-III follow-up, 72.6% of survivors survived without moderate or severe neurodevelopmental impairment, and 83.6% survived without severe impairment at 2–3 years’ corrected age.* • *Neonatal morbidity burden, rather than gestational age at rupture or latency duration, was more closely associated with later neurodevelopmental outcome, highlighting the importance of the postnatal clinical course for prognostication and counselling.*

**Supplementary information:**

The online version contains supplementary material available at 10.1007/s00431-026-07266-x.

## Introduction

Previable preterm prelabour rupture of membranes (pPROM) before 23 + 0 weeks’ gestation is a rare but severe obstetric complication, affecting approximately 0.1–0.7% of pregnancies [1–4]. At this stage, management is complex, as expectant care carries significant risks for both mother and foetus, including infection, foetal demise, pulmonary hypoplasia, limb deformities, and long-term morbidity [1, 4, 5]. As viability approaches, neonatal intervention becomes possible, requiring time-sensitive and complex decision-making. Counselling remains difficult due to limited and heterogeneous evidence. Reported neonatal survival after pPROM before 23 weeks’ gestation ranges from 23 to 95%, with survival without major morbidity ranging from 26 to 81% [6–16]. These variations reflect heterogeneity in study design, diagnostic criteria, and population characteristics.

Although several cohort studies have reported long-term outcomes after previable pPROM, the available evidence remains limited and heterogeneous. Neurodevelopmental outcomes vary considerably because studies differ substantially with regard to study design, inclusion criteria, gestational age at membrane rupture and delivery, duration of follow-up, and, importantly, the methods used to assess long-term outcome [2, 6, 15–21]. Consequently, reported rates of moderate-to-severe neurodevelopmental impairment range from approximately 16–48%, with gross motor and cognitive delays reported in 25–50% and speech delay in up to 77.5% [15–19]. Beyond early childhood, reported sequelae include behavioural disorders, reduced lung function, and echocardiographic signs of pulmonary hypertension [2, 6, 20]. Direct comparison between studies is therefore challenging.

Prospective, longitudinal studies using standardized neurodevelopmental assessments remain scarce. Until such data become available, well-characterized institutional cohorts with standardized follow-up protocols continue to provide valuable information for parental counselling and clinical decision-making [13, 15, 21, 22]

We previously reported short-term outcomes of 109 infants with pPROM < 23 + 0 weeks who received active care at our institution (2009–2022), with 69.7% survival [22]. This study now extends follow-up to evaluate long-term developmental and functional outcomes using standardized neurodevelopmental assessment, thereby addressing an important knowledge gap and supporting clinical decision-making at the limit of viability.

## Materials and methods

### Study setting

This retrospective study was conducted at a tertiary perinatal centre with 54 neonatal beds and an annual delivery volume of 2800 to 3000 births, caring for approximately 90 infants < 1000 g and 180 infants < 1500 g annually, with survival rates of 82% and 90%, respectively. Eligible cases were pregnancies with pPROM before 23 + 0 weeks of gestation in which active neonatal care was initiated; in twin pregnancies, only the foetus affected by pPROM was included in the analysis. The study covered June 1, 2009, to December 31, 2022, and excluded foetuses with chromosomal anomalies or major malformations. The Institutional Review Board approved the study (reference 1415/2014), and informed consent was waived due to its retrospective design.

### Obstetrical standard of care

The diagnosis of pPROM was based on visible amniotic fluid leakage or positive biochemical amniotic fluid tests. Antenatal management included corticosteroids, antibiotics, and magnesium sulphate since 2015. Further management details have been described previously [22]

### Delivery room management and short-term outcomes

Delivery room management included early high-flow continuous positive airway pressure, prophylactic surfactant via the less-invasive surfactant administration (LISA) technique, and empirical antibiotic therapy for at least 48 h [22]. Obstetric and perinatal data were extracted from medical records.

Mortality before discharge was recorded. Severe cerebral morbidity was defined as grade III intraventricular haemorrhage (IVH), periventricular haemorrhagic infarction (PVHI), or cystic periventricular leukomalacia (cPVL). Bronchopulmonary dysplasia (BPD) was defined as oxygen dependence at 36 weeks’ postmenstrual age, and severe retinopathy of prematurity (ROP) as stage ≥ 3. Additional outcomes included surgically treated persistent ductus arteriosus (PDA) and necrotizing enterocolitis (NEC) stage > 2 with surgical treatment. Severe morbidity was defined as a composite of severe cerebral morbidity, NEC, BPD, and/or severe ROP.

### Long-term outcomes

Neurodevelopmental follow-up was conducted at 24–36 months corrected age by a multidisciplinary team, including neonatologists and developmental psychologists. Standardized neurological examination and developmental assessment were performed using the Bayley Scales of Infant Development, third edition (Bayley-III). Cerebral palsy was diagnosed according to international criteria and classified according to the Gross Motor Function Classification System (GMFCS).

Based on German norms, outcomes were classified into four groups: normal (≥ 85), mild impairment (70–84), moderate impairment (55–69), and severe impairment (< 55). Moderate or severe neurodevelopmental impairment (NDI) was defined as Bayley-III cognitive score < 70, cerebral palsy GMFCS level II–V, bilateral blindness, or severe hearing loss. Severe NDI was defined as Bayley-III cognitive score < 55, cerebral palsy GMFCS level IV–V, bilateral blindness, or severe hearing loss.

All follow-up data were retrieved from our institutional electronic database and verified individually by chart review.

### Statistical analysis

Statistical analyses were performed using IBM SPSS Statistics for Mac, version 23.0 (IBM Corp., Armonk, NY, USA). Continuous variables were assessed for distribution using visual assessment of approximate normality (e.g., histograms) and are presented as mean ± standard deviation (SD) or median with interquartile range (IQR), as appropriate. Categorical variables are presented as counts and percentages. Group comparisons were performed using the Student’s t-test or Mann–Whitney U test for continuous variables and the χ^2^ test or Fisher’s exact test for categorical variables, as appropriate.

Neurodevelopmental outcomes were analysed both as continuous variables (Bayley-III scores) and as categorical outcomes (NDI categories). Subgroup analyses (e.g., latency duration and gestational age at pPROM) were conducted using predefined exploratory cut-offs based on prior literature (< 28 vs. ≥ 28 days; ≤ 20 vs. > 20 weeks).

Multivariable logistic regression was performed to identify factors independently associated with survival without moderate to severe NDI. Based on biological plausibility and to avoid overfitting, predictors were prespecified. A baseline model evaluated perinatal predictors (gestational age at birth, latency duration, and sex), whereas a separate prognostic model additionally included the number of major neonatal morbidities (IVH III, PVHI, cPVL, BPD, ROP). Due to the small sample size, additional variables were not included. Given the limited sample size and number of outcome events, model complexity was restricted to prespecified variables to minimize overfitting, and the regression analyses should be interpreted with caution as primarily hypothesis-generating. Results are presented as odds ratios (ORs) with 95% confidence intervals (CIs). No additional model diagnostics were performed. A two-sided p-value < 0.05 was considered statistically significant.

## Results

Data from 109 infants with pPROM < 23 + 0 weeks who received active care between June 2009 and December 2022 at our institution were analysed (Supplementary Fig. 1). Of these 109 infants, 33 died before discharge (30.3%). Among the 76 survivors, 13 (17.1%) were lost to follow-up, and 2–3-year outcome data were available for 63/76 survivors, representing an 82.9% follow-up rate among survivors. Maternal and neonatal characteristics of infants with and without follow-up are presented in Tables 1 and 2. Compared with infants included in follow-up, those lost to follow-up had higher cord blood pH, lower neonatal lactate concentrations, higher 10-min Apgar scores, and a higher rate of survival without severe neonatal morbidity.

**Table 1:** Maternal characteristics of pregnancies complicated by previable pPROM

	Survivors with outcome *n* = 63	Survivors without outcome *n* = 13
Maternal characteristics		
Maternal age (years)*	31.7 (27.9–35.8)	30.9 (27.6–33.9)
Maternal antibiotics, *n* (%)	63 (100.0)	13 (100.0)
Tocolytics, *n* (%)	63 (100.0)	13 (100.0)
Antenatal steroids, *n* (%)	63 (100.0)	13 (100.0)
C-section, *n* (%)	56 (88.9)	11 (84.6)
Maternal CRP, mg/dL^a^*	0.63 (0.31–1.73)	1.12 (0.29–2.42)
Maternal leukocytes, G/L^a^*	12.78 (10.15–15.27)	12.40 (10.45–14.86)
Normal amniotic fluid volume, *n* (%)	8 (12.9)	4 (30.8)
Oligohydramnios^b^, *n* (%)	24 (38.7)	3 (23.1)
Oligoanhydamnios/anhydramnios^b^, *n* (%)	30 (48.4)	6 (46.2)
Suspected clinical chorioamnionitis^b^, *n* (%)	15 (23.8)	1 (7.7)
Placental abruption, *n* (%)	2 (3.2)	1 (7.7)
Cord prolapse, *n* (%)	1 (1.6)	0 (0.0)
Maternal sepsis, *n* (%)	3 (4.8)	0 (0.0)
Positive placental culture^c^, *n* (%)	24 (51.1)	2 (22.2)

^*^Median (IQR)

^a^Last count before delivery

^b^Amniotic fluid status was classified from antenatal ultrasound reports as normal fluid volume, oligohydramnios, oligoanhydramnios, or anhydramnios. For the exploratory analysis, infants were grouped as normal fluid volume/oligohydramnios versus oligoanhydramnios/anhydramnios. Oligohydramnios was defined as an amniotic fluid index < 5 cm, and anhydramnios as a maximum vertical pocket < 1 cm following membrane rupture. Oligoanhydramnios was diagnosed according to the contemporaneous ultrasound report when fluid volume was considered intermediate between oligohydramnios and anhydramnios. Clinical chorioamnionitis was suspected in the presence of maternal fever > 38 °C, tachycardia > 100/min, purulent vaginal discharge, uterine tenderness, or fetal tachycardia > 160/min

^c^Placental and membrane cultures were obtained only in patients delivered by caesarean section

**Table 2:** Neonatal characteristics and neonatal short-term outcome

	Survivors with outcome *n* = 63	Survivors without outcome *n* = 13
Neonatal characteristics		
GA at pPROM (weeks)*	21.6 (20.6–22.4) min–max: 12.1–22.9	21.9 (21.4–22.3) min–max: 18.0–22.9
GA at birth (weeks)*	25.7 (24.0–26.6) min–max: 22.9–29.6	26.4 (25.4–27.4) min–max: 23.0–28.6
Latency time pPROM to birth (days)*	26 (17–44) min–max: 2–94	34 (23–41) min–max: 3–58
Sex (male, *n* (%))	41 (65.1)	5 (38.5)
Birthweight (g)°	814 ± 222 min–max: 432–1305	899 ± 209 min–max: 570–1200
Cord blood pH*	7.35 (7.29–7.39)	7.39 (7.34–7.46)
Cord blood pH < 7.0, *n* (%)	1 (1.9)	0 (0.0)
Neonatal pH*	7.17 (7.08–7.22)	7.21 (7.14–7.28)
Neonatal pH < 7.0, *n* (%)	6 (9.7)	0 (0.0)
Neonatal lactate (mmol/l)*	4.0 (3.3–6.1)	2.9 (2.0–3.9)
Neonatal lactate > 6, *n* (%)	15 (25.0)	0 (0.0)
APGAR 1 min/5 min/10 min*	7 (6–8)/8 (8–9)/9 (9–9)	8 (7–9)/9 (8–9)/9 (9–9)
APGAR 5 min < 5, *n* (%)	0 (0.0)	0 (0.0)
Neonatal morbidities		
EONS any, *n* (%)	6 (9.7)	0 (0.0)
Culture-positive	1 (1.6)	0 (0.0)
Culture-negative	5 (7.9)	0 (0.0)
PDA with surgical intervention, *n* (%)	5 (7.9)	0 (0.0)
NEC with surgical intervention, *n* (%)	8 (12.7)	1 (7.7)
IVH III/PVHI, *n* (%)	7 (11.1)	0 (0.0)
cPVL, *n* (%)	0 (0.0)	0 (0.0)
BPD (oxygen at 36 weeks gestational age), *n* (%)	21 (36.8)	5 (38.5)
ROP with intervention, *n* (%)	14 (22.2)	1 (7.7)
Survival without severe morbidity, *n* (%)	30 (47.6)	11 (84.6)

Severe morbidity: IVH grade III, PVHI, cPVL, NEC, BPD, and/or ROP

*GA* gestational age, *pPROM* preterm prelabour rupture of membranes, *EONS* early-onset neonatal sepsis (culture-positive and culture-negative following established criteria)

^*^Median (IQR)

°Mean ± SD

Tables 1 and 2 summarize the maternal and neonatal characteristics and short-term outcomes of the 63 survivors. Overall, pPROM occurred at a mean gestational age of 21.6 weeks. The mean gestational age at delivery was 25.7 weeks, mean birth weight was 814 g, and median latency was 26 days (range 2–94).

Thirty infants (47.6%) survived without severe short-term morbidity, and these infants had a significantly higher gestational age at birth and a higher birth weight compared to those who experienced severe short-term morbidity. Table 3 summarizes follow-up outcomes at 2–3 years’ corrected age. The median Bayley-III scores were 90 (IQR 73–100) for cognition, 81 (IQR 63–97) for language, and 89 (IQR, 65–100) for motor function. CP was diagnosed in 11.1%. Survival without moderate or severe NDI was observed in 72.6% of patients, and survival without severe NDI in 83.6%.

**Table 3:** Neurodevelopmental outcome in survivors stratified by latency duration, gestational age at pPROM, and presence of severe neonatal morbidity

	Survivors (*n* = 63)	Latency ≤ 28 (*n* = 33)	Latency > 28 (*n* = 30)	*p*-value	GA at pPROM ≤ 20 weeks (*n* = 19)	GA at pPROM > 20 weeks (*n* = 44)	*p*-value	No severe morbidity (*n* = 30)	Severe morbidity (*n* = 33)	*p*-value
GA at birth (weeks)*	25.71 (24.00–26.57)	24.14 (23.43–25.29)	26.64 (26.11–27.71)	** < 0.001**	26.29 (25.57–27.43)	25.29 (23.90–26.14)	**0.015**	26.14 (25.43–26.97)	24.71 (23.57–26.43)	**0.019**
Birth weight*	800 (610–940)	645 (567–835)	890 (800–1074)	** < 0.001**	880 (793–1185)	793 (588–925)	**0.023**	905 (795–1063)	712 (577–855)	**0.001**
Birth weight percentile*	51 (31–74)	52 (26–81)	51 (36–70)	0.863	54 (47–81)	48 (24–73)	0.164	66 (46–82)	47 (23–63)	**0.020**
Latency (days)*	26 (17–44)	17 (11–23)	44 (34–57)	** < 0.001**	51 (38–63)	23 (13–31)	** < 0.001**	32 (22–49)	23 (14–41)	0.090
GA at pPROM (weeks)*	21.57 (20.57–22.43)	22.29 (21.43–22.57)	20.79 (18.65–21.47)	** < 0.001**	20.00 (17.43–20.43)	22.14 (21.43–22.57)	** < 0.001**	21.50 (20.43–22.57)	21.57 (20.64–22.22)	0.730
Corrected age at follow-up (years)*	2.02 (2.00–2.05)	2.02 (2.00–2.07)	2.01 (2.00–2.05)	0.534	2.02 (2.00–2.05)	2.02 (1.99–2.06)	0.620	2.01 (1.98–2.03)	2.05 (2.00–2.12)	**0.003**
Cognitive outcome*	90 (73–100)	88 (66–100)	90 (78–103)	0.452	88 (68–110)	90 (73–100)	0.853	95 (85–105)	80 (59–95)	**0.027**
> 85, *n* (%)	33 (62.3)	15 (53.6)	18 (72.0)	0.272	11 (68.8)	22 (59.5)	0.740	21 (77.8)	12 (46.2)	**0.037**
84–70, *n* (%)	8 (15.1)	6 (21.4)	2 (8.0)		1 (6.3)	7 (18.9)		3 (11.1)	5 (19.2)	
69–55, *n* (%)	4 (7.5)	2 (7.1)	2 (8.0)		2 (12.5)	2 (5.4)		1 (3.7)	3 (11.5)	
< 55, *n* (%)	8 (15.1)	5 (17.9)	3 (12.0)		2 (12.5)	6 (16.2)		2 (7.4)	6 (23.1)	
Language outcome*	81 (63–97)	77 (60–97)	84 (69–99)	0.353	91 (61–110)	78 (63–91)	0.077	87 (69–97)	74 (59–93)	0.127
> 85, *n* (%)	23 (43.4)	11 (39.3)	12 (48.0)	0.777	11 (68.8)	12 (32.4)	0.685	15 (55.6)	8 (30.8)	0.495
84–70, *n* (%)	13 (24.5)	8 (28.6)	5 (20.0)		1 (6.3)	12 (32.4)		5 (18.5)	8 (30.8)	
69–55, *n* (%)	8 (15.1)	3 (10.7)	5 (20.0)		1 (6.3)	7 (18.9)		4 (14.8)	4 (15.4)	
< 55, *n* (%)	9 (17.0)	6 (21.4)	3 (12.0)		3 (18.8)	6 (16.2)		3 (11.1)	6 (23.1)	
Motor outcome*	89 (65–100)	85 (60–98)	89 (69–100)	0.577	89 (76–96)	85 (62–103)	0.901	96 (85–104)	75 (55–92)	** < 0.001**
> 85, *n* (%)	36 (60.0)	20 (62.5)	16 (57.1)	0.874	11 (61.1)	25 (59.5)	0.863	25 (83.3)	11 (36.7)	**0.001**
84–70, *n* (%)	9 (15.0)	3 (9.4)	6 (21.4)		4 (22.2)	5 (11.9)		3 (10.0)	6 (20.0)	
69–55, *n* (%)	8 (13.3)	3 (9.4)	5 (17.9)		2 (11.1)	6 (14.3)		2 (6.7)	6 (20.0)	
< 55, *n* (%)	7 (11.7)	6 (18.8)	1 (3.6)		1 (5.6)	6 (14.3)		0 (0.0)	7 (23.3)	
CP any, *n* (%)	7 (11.1)	5 (15.2)	2 (6.7)	0.429	1 (5.3)	6 (13.6)	0.427	0 (0.0)	7 (21.2)	**0.011**
CP ≥ II, *n* (%)	5 (7.9)	3 (9.1)	2 (6.7)	1.000	1 (5.3)	4 (9.1)	0.678	0 (0.0)	5 (15.2)	0.054
Ambulant CP (GMFCS level I-II), *n* (%)	3 (4.8)	2 (6.1)	1 (3.3)	1.000	1 (5.3)	2 (4.5)	1.000	0 (0.0)	3 (9.1)	0.240
Non-ambulant CP (GMFCS level III-V), *n* (%)	4 (6.3)	3 (9.1)	1 (3.3)	0.614	0 (0.0)	4 (9.1)	0.306	0 (0.0)	4 (12.1)	0.115
Visual impairment, *n* (%)	7 (11.1)	6 (18.2)	1 (3.3)	0.107	2 (10.5)	5 (11.4)	1.000	0 (0.0)	7 (21.2)	**0.011**
Mild (glasses), *n* (%)	3 (4.8)	3 (9.1)	0 (0.0)		1 (5.3)	2 (4.5)		0 (0.0)	3 (9.1)	
Moderate/severe, *n* (%)	4 (6.3)	3 (9.1)	1 (3.3)		1 (5.3)	3 (6.8)		0 (0.0)	4 (12.1)	
Hearing impairment, *n* (%)	0 (0.0)	0 (0.0)	0 (0.0)	NA	0 (0.0)	0 (0.0)	NA	0 (0.0)	0 (0.0)	NA
Survival at 2–3 years with Bayley score ≥ 70, *n* (%)	36 (58.1)	20 (62.5)	16 (53.3)	0.465	12 (63.2)	24 (55.8)	0.589	21 (72.4)	15 (45.5)	**0.032**
Survival without moderate or severe NDI (< 70 cognitive, hearing or visual loss, or CP level II-V), *n* (%)	45 (72.6)	21 (65.6)	24 (80.0)	0.205	14 (73.7)	31 (72.1)	0.897	26 (89.7)	19 (57.6)	**0.005**
Survival at 2–3 years with Bayley score > 55, *n* (%)	45 (76.3)	23 (74.2)	22 (78.6)	0.693	15 (78.9)	30 (75.0)	0.761	24 (82.8)	21 (70.0)	0.249
Survival without severe NDI (< 55 cognitive, hearing or visual loss, or CP level ≥ IV), *n* (%)	51 (83.6)	26 (78.8)	25 (89.3)	0.319	17 (89.5)	34 (81.0)	0.485	28 (93.3)	23 (74.2)	0.081

Severe morbidity: IVH grade III, PVHI, cPVL, NEC, BPD, and/or ROP

*GA* gestational age, *pPROM* preterm prelabour rupture of membranes, *CP* cerebral palsy, *GMFCS* Gross Motor Function Classification System, *NDI* neurodevelopmental impairment

^*^Median (IQR)

Data stratified by latency (< 28 vs. ≥ 28 days) or by gestational age at pPROM (≤ 20 vs. > 20 weeks) were not associated with significant differences in neurodevelopmental outcomes. Stratification according to the worst documented amniotic fluid status (normal/oligohydramnios vs. oligoanhydramnios/anhydramnios) showed a trend toward higher rates of BPD among infants with severe reduction of amniotic fluid but no association with long-term neurodevelopmental outcomes (Supplementary Table 1). Stratification by severe short-term morbidity showed significantly lower cognitive and motor scores and higher rates of CP and visual impairment. Correspondingly, survival without moderate or severe NDI was significantly lower among affected infants (57.6%) compared with those without severe short-term morbidity (89.7%).

Table 4 summarizes the multivariable logistic regression models for survival without moderate or severe NDI. In the baseline logistic regression model including perinatal predictors only, higher gestational age at birth was associated with increased odds of the outcome (OR 1.88, 95% CI 1.02–3.46, *p* = 0.043), and female sex was also independently associated (OR 6.88, 95% CI 1.36–34.83, *p* = 0.020). Latency duration showed no association (*p* = 0.950). After addition of the number of major neonatal morbidities, gestational age at birth and sex were no longer associated with the outcome, whereas a higher morbidity burden remained independently associated (OR 0.39, 95% CI 0.17–0.89, *p* = 0.025).

**Table 4:** Multivariable logistic regression models identifying factors associated with survival without moderate or severe neurodevelopmental impairment

		*p*-value	OR	95% CI
Model 1	GA at birth (weeks)	**0.043**	**1.878**	**1.019–3.463**
	Latency (days)	0.950	1.001	0.960–1.045
	Sex	**0.020**	**6.882**	**1.360–34.830**
Model 2	GA at birth (weeks)	0.307	1.430	0.720–2.840
	Latency (days)	0.948	1.002	0.952–1.054
	Sex	0.092	4.259	0.789–22.980
	Number of severe morbidities	**0.025**	**0.388**	**0.170–0.887**

Number of major neonatal morbidities: IVH III, PVHI, cPVL, NEC, BPD, and ROP. Model 1: gestational age at birth, latency duration, and sex (reference category: male). Model 2: Model 1 plus number of major neonatal morbidities. Severe morbidity: IVH grade III, PVHI, cPVL, NEC, BPD, and/or ROP

*GA* gestational age, *OR* odds ratio

## Discussion

In this single-centre cohort of infants with pPROM before 23 weeks’ gestation receiving active care, survival without moderate or severe NDI was observed in 72.6% of patients, and survival without severe NDI in 83.6%. These findings suggest that meaningful survival without major impairment is achievable in this highly selected population despite the extreme antenatal risk associated with very early membrane rupture.

Direct comparison with the limited existing literature reporting standardized neurodevelopmental follow-up after extremely early pPROM is, however, difficult because differences in outcome assessment may substantially affect impairment classification. In the large registry analysis by Young et al. (*n* = 690), survival to discharge was 73% with 19.2% severe and 46.3% moderate or severe NDI [16], whereas Pendse reported survival of 79.3% with moderate impairment in 25% and no severe impairment in a smaller cohort (*n* = 82) [15]. These comparisons should be interpreted with caution, as our Bayley-III assessments were based on German norms, which may classify developmental performance more stringently than the standard US norms used in many international studies and could influence the categorization of impairment severity. Differences in impairment classification related to assessment tools and normative data have been described previously [23]. Direct comparison with infants born preterm following different pathways to preterm birth should be interpreted cautiously, as accumulating evidence suggests that distinct pathophysiological pathways to preterm birth are associated with different neonatal phenotypes and outcome patterns [24, 25].

A central observation of our study is that antenatal variables traditionally considered prognostic, including gestational age at pPROM and latency duration, were not independently associated with NDI among survivors.

This is consistent with emerging evidence suggesting that their primary impact relates to perinatal survival rather than later neurodevelopment. A well-documented mortality gradient with earlier rupture has been reported across multiple cohorts [2, 6, 15, 16, 22, 26], while associations with severe neonatal morbidity remain heterogeneous but generally indicate increased risk of short-term morbidities following very early pPROM [2, 6, 22].

In contrast, available long-term data remain limited and inconsistent: in smaller cohort studies, including ours and that of Pendse et al., no clear association between rupture timing and neurodevelopment among survivors was observed [15], whereas larger registry-based analyses have identified a significant association [16]. The trend toward higher language scores in the subgroup with rupture before 20 weeks in our cohort likely reflects the higher gestational age at birth in this subgroup (Table 3).

The lack of an independent association between latency duration and long-term outcomes is notable given the long-standing clinical perception that prolonged latency after very early pPROM may both enhance foetal maturation and increase the risks of infection and pulmonary hypoplasia. This finding is, however, biologically plausible when considered in the context of interdependence between latency and gestational age at rupture. In our cohort, longer latency translated into higher gestational age at birth (Table 3), consistent with the well-established inverse relationship between rupture timing and achievable latency [6, 15, 27]. Accordingly, latency represents a mechanism through which gestational age at birth is modified and short-term prognosis is improved [6, 22]. For long-term neurodevelopment, however, this close interdependence likely precludes latency from acting as an independent determinant. Our results therefore support the interpretation that latency primarily operates as a mediator of perinatal maturation rather than a direct driver of neurodevelopmental outcome among survivors [16, 27].

Instead, the postnatal clinical course, particularly the burden of severe short-term neonatal morbidity, was more closely associated with later neurodevelopment in the prognostic model than antenatal timing variables. Severe short-term neonatal morbidity was closely associated with lower gestational age and birth weight, highlighting that higher gestational age was associated with lower rates of short-term morbidity, which in turn was associated with a lower risk of NDI (Table 3).

Importantly, almost half of the cohort survived without severe short-term morbidity, and these infants demonstrated substantially more favourable neurodevelopmental outcomes, including higher cognitive and motor scores, lower rates of cerebral palsy and visual impairment, and markedly higher survival without moderate to severe NDI (89.7% vs. 57.6%). This association has also been reported in other studies examining this specific relationship [2, 6]. This pattern is biologically plausible, as major neonatal complications such as severe brain injury, BPD, or advanced retinopathy represent final common pathways linking extreme prematurity and intrauterine adversity with long-term functional impairment. Our findings therefore support the concept that early antenatal events primarily influence the risk of preterm birth and neonatal instability, whereas neurodevelopmental trajectories appear to be more strongly associated with neonatal morbidity.

From a counselling perspective, our findings emphasize that prognostic discussions at the time of pPROM must occur under conditions of substantial uncertainty. While gestational age at rupture defines the biological starting point and frames the immediate risk, our data suggest that antenatal characteristics alone are insufficient to reliably predict longer-term outcomes. In the baseline model, gestational age at birth and sex were significant predictors, consistent with their established role as major determinants of early outcome [2, 16, 22]. However, after inclusion of neonatal morbidity burden, these associations weakened and lost statistical significance. This may partly reflect collinearity between gestational age at birth and neonatal complications, which are biologically and clinically closely linked, with complications representing downstream manifestations of underlying vulnerability. Accordingly, once the postnatal disease trajectory is established, neonatal morbidity burden may represent a more proximal predictor of later outcomes than maturational characteristics alone. Nevertheless, neonatal morbidity may itself represent an intermediate step through which antenatal factors influence long-term outcome, and therefore, an indirect effect of antenatal characteristics cannot be excluded.

### Strengths and limitations

Several limitations merit consideration. The single-centre design and relatively small sample size limit generalizability and statistical power, particularly for detecting modest antenatal effects. Importantly, outcome analyses were restricted to survivors, which may result in an overestimation of favourable neurodevelopmental outcomes due to survival bias. In addition, loss to follow-up among survivors introduces potential selection bias. Comparison of infants with and without follow-up suggested that those lost to follow-up experienced a somewhat less complicated neonatal course, raising the possibility that favourable long-term outcomes may be slightly underestimated.

Furthermore, our study was based exclusively on a NICU database and therefore included only infants who remained in utero after previable pPROM and subsequently received active neonatal care. The broader obstetric population—including pregnancies ending in stillbirth or managed with compassionate care before neonatal admission—was not captured. Consequently, our cohort represents a highly selected population, introducing additional selection bias and limiting the generalizability of our findings regarding overall perinatal outcomes. Subgroup analyses were limited by small sample sizes and may have been underpowered to detect clinically relevant differences. In addition, the limited number of outcome events constrained multivariable analyses, and regression results should be interpreted as hypothesis-generating. Finally, the retrospective design may be subject to residual confounding and unmeasured variables that could influence both neonatal morbidity and long-term outcomes. Despite these limitations, the study provides clinically relevant longitudinal outcome data in a rare and challenging population and contributes to a better understanding of the relationship between antenatal characteristics, neonatal morbidity, and early childhood neurodevelopment following extremely early pPROM.

Strengths of the study include the well-characterized, homogeneous single-centre cohort of survivors after previable pPROM, standardized neurodevelopmental follow-up using Bayley-III assessment, the high follow-up rate among survivors (82.9%), and the relatively large sample size for this rare condition. Together, these strengths allow a more consistent assessment of long-term neurodevelopmental outcomes than has been possible in many previous heterogeneous reports.

## Conclusion

In this cohort of infants with previable pPROM receiving active neonatal care, a substantial proportion of survivors achieved favourable neurodevelopmental outcomes at 2–3 years. Antenatal factors such as gestational age at rupture and latency duration were not independently associated with outcome among survivors, whereas neonatal morbidity burden was more closely associated with later neurodevelopmental outcome. These findings highlight the importance of considering the postnatal clinical course in addition to antenatal characteristics when counselling families at the limit of viability.

## Supplementary information

Below is the link to the electronic supplementary material.

ESM 1 (DOCX 221 KB)

## Data Availability

The data supporting the findings of this study are available within the article and its supplementary material. Additional de-identified individual participant data may be made available by the corresponding author upon reasonable request, subject to institutional, ethical, and data protection requirements.

## Abbreviations

BPD: Bronchopulmonary dysplasia
CI: Confidence interval
cPVL: Cystic periventricular leukomalacia
GMFCS: Gross Motor Function Classification System
IQR: Interquartile range
IVH: Intraventricular haemorrhage
LISA: Less-invasive surfactant administration
NEC: Necrotizing enterocolitis
NDI: Neurodevelopmental impairment
OR: Odds ratio
PDA: Persistent ductus arteriosus
pPROM: Preterm prelabour rupture of membranes
PVHI: Periventricular haemorrhagic infarction
ROP: Retinopathy of prematurity
SD: Standard deviation
