# Supplementary material for: Long-term outcomes after previable pPROM: implications for counselling at the limit of viability
Source: Eur J Pediatr. 2026 Jul 30;185(8):628. doi: 10.1007/s00431-026-07266-x (PMC13424032; doi:10.1007/s00431-026-07266-x)

**Supplementary Table 1.** Long-term neurodevelopmental outcomes and bronchopulmonary dysplasia according to the worst documented amniotic fluid status during pregnancy

| Outcome | Normal/oligohydramnios | Oligoanhydramnios/anhydramnios | p-value |
| --- | --- | --- | --- |
| BPD (oxygen at 36 weeks gestational age) n (%) | 6 (21.4) | 14 (50.0) | 0.050 |
| CP any n (%) | 4 (12.5) | 3 (10.0) | 1.000 |
| Survival without severe morbidity n (%) | 17 (53.1) | 13 (43.3) | 0.441 |
| Survival without moderate or severe NDI (<70 cognitive, hearing or visual loss, or CP level II-V) n (%) | 22 (71.0) | 22 (73.3) | 0.837 |
| Survival without severe NDI (<55 cognitive, hearing or visual loss, or CP level ≥IV) n (%) | 24 (77.4) | 26 (89.7) | 0.302 |

**Abbreviations:** BPD: bronchopulmonary dysplasia; CP: cerebral palsy; NDI: neurodevelopmental impairment. **Severe morbidity:** IVH grade III, PVHI, cPVL, NEC, BPD, and/or ROP.

**Supplementary Figure 1.** Study flow diagram


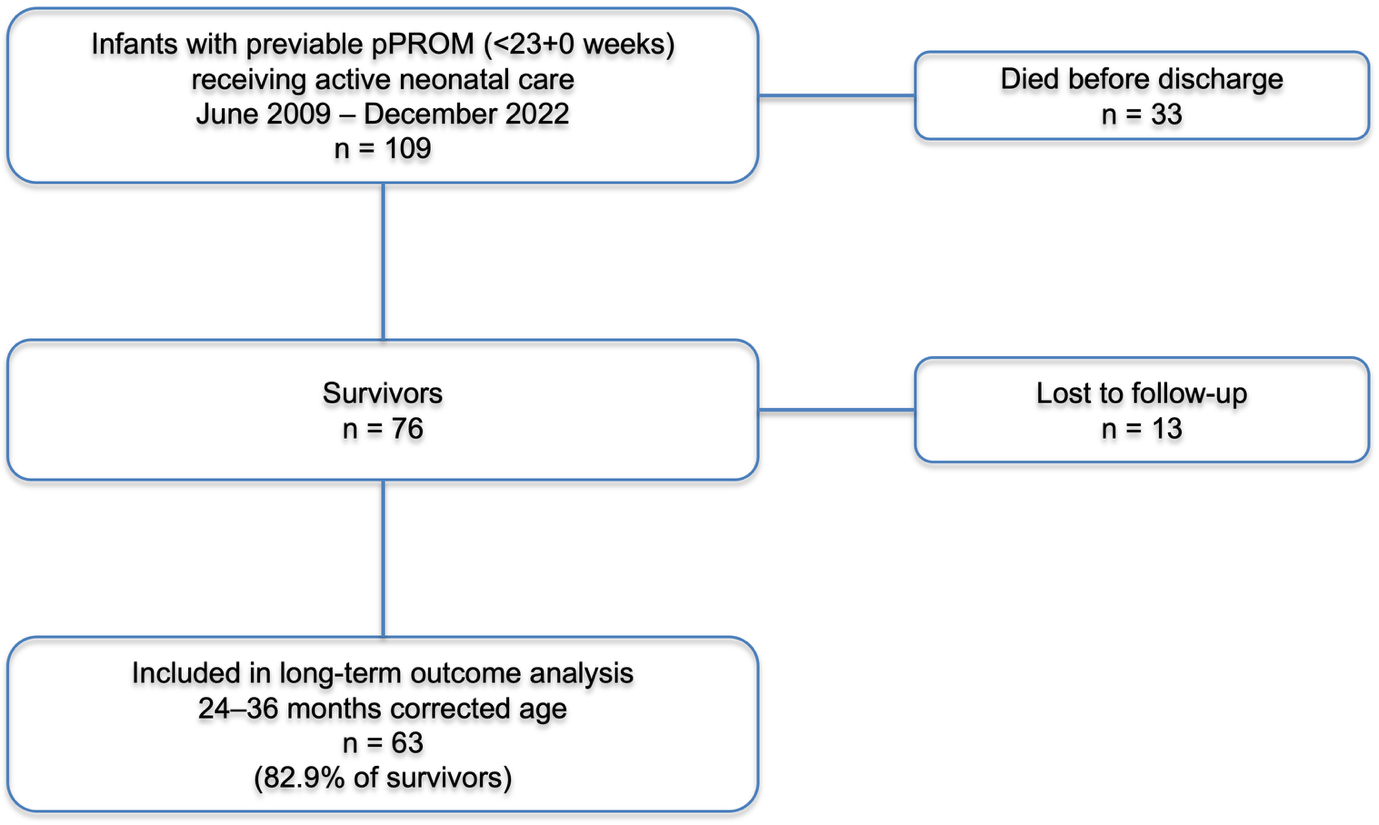

Supplement: Supplementary file 1 — (DOCX 221 KB) [file 431_2026_7266_MOESM1_ESM.docx]
